# Stillbirth in relation to maternal country of birth and other migration related factors: a population-based study in Norway

**DOI:** 10.1186/s12884-018-2140-3

**Published:** 2019-01-05

**Authors:** Eline S. Vik, Vigdis Aasheim, Erica Schytt, Rhonda Small, Dag Moster, Roy M. Nilsen

**Affiliations:** 1 Faculty of Health and Social Sciences, Western Norway University of Applied Sciences, Campus Kronstad, Inndalsveien 28, 5063 Bergen, Norway; 2 Department of Global Public Health and Primary Care, University of Bergen, Bergen, Norway; 3 Centre for Clinical Research Dalarna, Uppsala University, Uppsala, Sweden; 4 Department of Women’s and Children’s Health, Karolinska Institutet, Stockholm, Sweden; 5 Judith Lumley Centre, La Trobe University, Melbourne, Australia; 6 Department of Pediatrics, Haukeland University Hospital, Bergen, Norway

**Keywords:** Stillbirth, Migrant, Maternal country of birth, Paternal origin, Length of residence, Reason for immigration, Register study

## Abstract

**Background:**

Migrant women’s overall increased risk of adverse pregnancy outcomes is well known. The aim of this study was to investigate possible associations between stillbirth and maternal country of birth and other migration related factors (paternal origin, reason for immigration, length of residence and birthplace of firstborn child) in migrant women in Norway.

**Methods:**

Nationwide population-based study including births to primiparous and multiparous migrant women (*n* = 198,520) and non-migrant women (n = 1,156,444) in Norway between 1990 and 2013. Data from the Medical Birth Registry of Norway and Statistics Norway. Associations were investigated by multiple logistic regression and reported as odds ratios (ORs) with 95% confidence intervals (CIs).

**Results:**

Primiparous women from Sri-Lanka and Pakistan, and multiparous women from Pakistan, Somalia, the Philippines and Former Yugoslavia had higher odds of stillbirth when compared to non-migrant women (adjusted OR ranged from 1.58 to 1.79 in primiparous and 1.50 to 1.71 in multiparous women). Primiparous migrant women whose babies were registered with Norwegian-born fathers had decreased odds of stillbirth compared to migrant women whose babies were registered with foreign-born fathers (aOR = 0.73; CI 0.58–0.93). Primiparous women migrating for work or education had decreased odds of stillbirth compared to Nordic migrants (aOR = 0.58; CI 0.39–0.88). Multiparous migrant women who had given birth to their first child before arriving in Norway had higher odds of stillbirth in later births in Norway compared with multiparous migrant women who had their first child after arrival (aOR = 1.28; CI 1.06–1.55). Stillbirth was not associated with length of residence in Norway.

**Conclusions:**

This study identifies sub-groups of migrant women who are at an increased risk of stillbirth, and highlights the need to improve care for them. More attention should be paid to women from certain countries, multiparous women who had their first baby before arrival and primiparous women whose babies have foreign-born fathers.

**Electronic supplementary material:**

The online version of this article (10.1186/s12884-018-2140-3) contains supplementary material, which is available to authorized users.

## Background

Migrant women constitute a significant and growing proportion of childbearing women in high-income countries [1], and in 2016, 27% of all births in Norway were to migrant women [2]. An increased risk of several adverse pregnancy outcomes, such as low birth weight, preterm birth, congenital malformations, and perinatal morbidity and mortality has been found for some migrant women [3].

Stillbirth is associated with a wide range of health related risk factors including socioeconomic factors (high and low maternal age, low level of education and income), physical health problems (obesity, diabetes, hypertension, infections, drug use, smoking), obstetric history (primiparous, grand-multiparous, previous stillbirth), pregnancy complications (placenta dysfunction, preeclampsia, asphyxia, congenital anomalies), consanguinity, lack of antenatal care [4, 5] and the baby having a migrant father also seems to increase the risk of stillbirth [6]. Risk factors such as obesity and smoking are priorities for stillbirth prevention in high-income countries [5], while infections (including syphilis and HIV) and grand-multiparity are more frequently reported as causes of stillbirth in low- and middle-income countries [4].

Migrants constitute a diverse group. While refugees are likely to have been exposed to a range of health risks, others may be in better health, something which made migration possible (i.e. the healthy migrant effect) [7]. However, the healthy migrant effect does not apply to all migrants and health status deteriorates by length of residence for many [8]. The literature regarding migration and the risk of stillbirth is extensive, but the results are inconclusive [3, 7, 9] possibly due to heterogeneity of study designs and study samples, small numbers of women representing each country, and differences in the definition of migrants [9]. Most epidemiologic studies on stillbirth lack information on specific migration related factors [9]. In Norway, such information is registered for migrants, and available for research and surveillance purposes.

The aim of this study was to investigate possible associations between stillbirth and maternal country of birth and other migration related factors (paternal origin, reason for immigration, length of residence and birthplace of firstborn child) in migrant women in Norway.

## Methods

### Study design

This is a nationwide population-based study using data from the Medical Birth Registry of Norway (MBRN) and Statistics Norway (SSB). The MBRN is based on mandatory notification of all births in Norway since 1967 [10], and includes information on the pregnancy and the health of the mother and infant. SSB provides information on immigration and socioeconomic factors [11]. Data from MBRN and SSB were linked using each woman’s unique personal identification number.

### Setting

The health care system in Norway provides high quality care, antenatal and obstetric care is free of charge for all, and the risk of adverse neonatal health outcomes is in general low [12]. Antenatal care is provided either by general practitioners or midwives depending on the individual woman’s choice and medical needs and compliance with care is high [13]. However, migrant women make fewer visits and may not follow given recommendations to the same extent as non-migrants [14]. The vast majority of women in Norway give birth in hospitals (99%) [15].

In Norway, immigration has mainly been linked to growing labour demand, family reunion and refugees fleeing war and political conflicts. Migrants in Norway are more likely to have lower levels of education and be unemployed compared to the host population. They also have lower incomes, especially migrants from the African continent [11]. Every patient’s right to receive information suited to their age, language and culture is protected by law in Norway [16], yet an underuse of interpreting services by health care professionals has been reported, and family members or other unqualified individuals are often used as interpreters [17].

### Study population

**Fig. 1:**
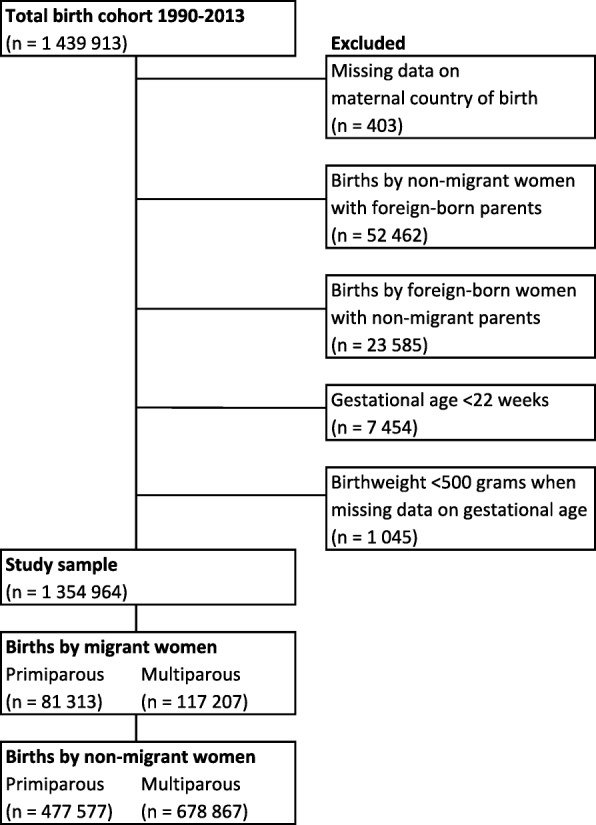
Derivation of the study sample. Flowchart

The total birth cohort from 1990 to 2013 included 1,439,913 births (Fig. 1). Exclusions were made to reduce the heterogeneity within the groups and compare births to migrant women who had non-Norwegian-born parents with births to non-migrants with Norwegian-born parents. We therefore excluded births with missing data on maternal country of birth, births to Norwegian-born women with at least one foreign-born parent and women born abroad with at least one Norwegian-born parent. We also excluded pregnancies if the gestational age was < 22 weeks or the infant birthweight was < 500 g (if missing data on gestational age) to conform with the definition of stillbirth (see below), leaving 1,354,964 singleton and multiple births for analyses.

### Stillbirth

Stillbirth was defined as a pregnancy loss at ≥22 weeks of gestation or with a birthweight ≥500 g if data on gestational age were missing [18]. To base the definition primarily on gestational age is considered appropriate as it includes more cases and predicts the maturity of the fetus and does not exclude fetuses suffering from growth restriction [18, 19].

### Migration related factors

Specific maternal countries of birth are reported for countries represented by a minimum of 6000 births (12 countries, Norway included), or by a stillbirth frequency of ≥20 throughout the study period (another 5 countries added), i.e. 17 countries altogether. The remaining countries were combined into *other countries* (34.7% of the births to migrant women). *Other countries* includes births to women from 177 different countries, which were categorized according to the Global Burden of Disease definitions [20]: Central Europe + Eastern Europe + Central Asia (16%), High-income (38%), Latin America + Caribbean (8%), North Africa + Middle East (8%), South Asia (5%), Sub-Saharan Africa (17%), Southeast Asia + East Asia + Oceania (6%) and Other (2%). Former Yugoslavia includes the following: Croatia, Slovenia, Bosnia and Herzegovina, Macedonia, Serbia, Montenegro and Kosovo.

Paternal country of birth was categorised into two groups: foreign-born and Norwegian-born. Data on paternal country of birth was missing in 5.0% of births to primiparous migrant women, and 10.3% of births to multiparous migrant women. The corresponding proportions of missing in primiparous and multiparous non-migrant women were 1.7 and 4.0%, respectively. Missing paternal country of birth may occur for various reasons and does not necessarily mean that the father was unknown.

Migrant women were grouped according to reasons for immigration to Norway using the categories *work/education, family (reunion or establishment), refuge,* and *unspecified/other* [21]. Nordic citizens may move freely to Norway without reporting their reason for immigration and were therefore categorized into a separate exposure group (*Nordic migrants*)*.* Work and education are related reasons for immigration and were combined due to small numbers.

The mother’s length of residence (in years) was calculated as the difference between the year of delivery and the year of official permission to stay in Norway. Most migrants from outside the European Union/European Economic Area will have received a residence permit before entering Norway. The exception is those applying for asylum who will have received a decision within 6 months of applying [22]. Birthplace of multiparous women’s first child (*Norway*, *Other than Norway*) was assessed by their parity registered in the MBRN. If a woman’s first birth in the MBRN was registered with parity 0, the birthplace of her first child was in Norway. If a woman’s first birth in the MBRN was registered with parity 1 or higher, the birthplace of her first child was outside Norway.

Information on *reason for immigration* was only available from 1990 onwards. Further, due to data truncation we did not have information on previous pregnancies in migrant women coming to Norway before 1990. Therefore, women who received permission to stay in Norway before 1990, but gave birth from 1990 onwards, were excluded from the analyses when investigating the association with stillbirth of *reason for immigration* (*n* = 34,303 births excluded) and *birthplace of firstborn child* (*n* = 23,890 births to multiparous migrant women excluded).

### Other variables

From the MBRN we obtained data on year of birth, maternal age (< 25, 25–29, 30–34, 35–39, ≥40), marital status (married/cohabiting, not married/cohabiting), consanguinity (second cousin or closer, not related), chronic hypertension (yes, no), pre-eclampsia/eclampsia (yes, no), pre-pregnancy diabetes (yes, no), maternal overweight (BMI ≥ 25), smoking before pregnancy (yes, no), parity (0, 1, 2, 3, 4, ≥5), gestational age (very preterm (22–27 weeks), moderately preterm (28–36 weeks), term (37–41 weeks), or post term birth (≥ 42 weeks)) and recurrent stillbirth (yes, no). For each birth year SSB provided data on mother’s gross income (categorised into quartiles) and level of education (no education, primary school, secondary school, university/college).

### Statistics

The analyses were performed for primiparous and multiparous women separately, as these groups are managed differently in clinical guidelines in antenatal care [23].

To investigate the association between maternal country of birth and other immigration related factors and stillbirth, we estimated crude and adjusted odds ratios (aOR) with 95% confidence intervals (CI) using logistic regression analysis. Adjustments were made for year of birth, parity, maternal age, marital status, mother’s income, level of education and consanguinity. Country of birth and the other immigration related factors were investigated one at a time and not mutually adjusted. To account for dependency between births to the same mother, we used robust standard errors that allowed for within-mother clustering [24].

Information on education and income was missing in 4 and 13% of the total sample (25 and 42% in the migrant group), respectively. To avoid discarding valuable data in adjusted regression analyses, a multiple imputation technique was used to replace missing values assumed to be *missing at random.* The imputation algorithm used was multivariate normal [25], and a total number of 10 imputed datasets were created. The imputation model used for analyses of maternal country of birth included stillbirth, maternal country of birth, year of birth, parity, maternal age, marital status, consanguinity, education and income. The imputation models used for analyses of each of the other migration related factors also included paternal origin, reason for immigration, length of residence, or birthplace of firstborn child.

In the analyses of maternal country of birth, the non-migrant women were defined as the reference group. For other migration related variables (paternal origin, reason for immigration, length of residence and birthplace of firstborn child), the most common category among migrants was chosen as the reference, non-migrant women excluded.

All analyses were performed using Stata IC version 14 (Stata Statistical Software, College Station, TX, USA) and Statistical Package for Social Science version 23 (SPSS Inc., Chicago, IL, USA).

## Results

Characteristics of the study sample are shown in Table 1. Primiparous migrant women were more likely to be older (> 35 years), single, and to have a lower level of education and income, and less likely to be < 25 years, to be overweight or to smoke compared to primiparous non-migrant women. Multiparous migrant women were more likely to be older, to have a lower level of education and income, to have diabetes type 2 and to have more children than multiparous non-migrant women; however, they were less likely to smoke. Parental consanguinity was also more common in some migrant women (Table 1). Consanguinity was more common among migrant women from Pakistan (primiparous 25.8%, multiparous 26.3%) and Turkey (primiparous 11.6%, multiparous 11.3%), compared to non-migrant women (primiparous 0.1%, multiparous 0.2%) (not shown in tables).

**Table 1:** Maternal characteristics by migrant and non-migrant women giving birth in Norway, 1990–2013^a^

	Primiparous women		Multiparous women	
	Migrant	Non-migrant	Migrant	Non-migrant
	(*n* = 80,119)	(*n* = 468,983)	(*n* = 115,606)	(*n* = 667,654)
	*n (%)*	*n (%)*	*n (%)*	*n (%)*
Age (years)				
< 25	23,983 (29.9)	163,323 (34.8)	12,629 (10.9)	63,940 (9.6)
25–29	29,379 (36.7)	180,607 (38.5)	34,481 (29.8)	212,756 (31.9)
30–34	19,392 (24.2)	93,888 (20.0)	40,957 (35.4)	254,405 (38.1)
35–39	6338 (7.9)	26,923 (5.7)	22,501 (19.5)	116,858 (17.5)
≥40	1027 (1.3)	4242 (0.9)	5038 (4.4)	19,695 (2.9)
Single status ^b^	6652 (8.3)	58,059 (12.4)	8864 (7.7)	34,807 (5.2)
Consanguinity, second cousin or closer	2082 (2.6)	594 (0.1)	4467 (3.9)	1167 (0.2)
Mother’s education				
No education	856 (1.1)	4 (0.0)	3541 (3.1)	41 (0.0)
Primary education	15,538 (19.4)	96,802 (20.6)	30,960 (26.8)	147,867 (22.1)
Secondary school	15,897 (19.8)	180,494 (38.5)	23,811 (20.6)	255,916 (38.3)
University/college	26,002 (32.5)	190,883 (40.7)	29,978 (25.9)	262,404 (39.3)
Missing	21,826 (27.2)	800 (0.2)	27,316 (23.6)	1426 (0.2)
Mother’s income^c^				
≤25 percentile	18,250 (22.8)	98,494 (21.0)	26,044 (22.5)	146,471 (21.9)
25–50 percentile	12,391 (15.5)	98,342 (21.0)	16,943 (14.7)	163,221 (24.4)
50–75 percentile	8793 (11.0)	121,703 (26.0)	10,600 (9.2)	148,630 (22.3)
≥75 percentile	9898 (12.4)	122,450 (26.1)	11,149 (9.6)	142,284 (21.3)
Missing	30,787 (38.4)	27,994 (6.0)	50,870 (44.0)	67,048 (10.0)
Chronic hypertension	245 (0.3)	2043 (0.4)	518 (0.4)	3506 (0.5)
Pre-eclampsia/eclampsia	2971 (3.7)	25,391 (5.4)	2252 (1.9)	15,988 (2.4)
Pre-pregnancy diabetes ^d^				
Type 1	152 (0.2)	1616 (0.3)	314 (0.3)	2057 (0.3)
Type 2	157 (0.2)	402 (0.1)	476 (0.4)	746 (0.1)
Maternal overweight (BMI ≥ 25) ^e^	3501 (10.4)	17,673 (16.1)	7454 (16.5)	26,520 (17.7)
Not overweight	12,622 (37.5)	33,785 (30.8)	12,887 (28.5)	42,331 (28.2)
Missing	17,548 (52.1)	58,371 (53.1)	24,952 (55.1)	81,359 (54.2)
Smoking before pregnancy ^e^	3133 (5.0)	21,990 (7.9)	3014 (3.4)	22,603 (5.7)
Non-smoker	21,343 (33.7)	67,540 (24.4)	29,655 (33.3)	104,803 (26.6)
Missing	38,807 (61.3)	187,681 (67.7)	56,414 (63.3)	267,054 (67.7)
Former stillbirths			2639 (2.3)	9336 (1.4)
Parity				
0	80,119 (100.0)	468,983 (100.0)		
1			64,191 (55.5)	411,085 (61.6)
2			29,859 (25.8)	189,681 (28.4)
3			12,127 (10.5)	48,959 (7.3)
4			5043 (4.4)	11,999 (1.8)
≥5			4386 (3.8)	5930 (0.9)
Gestational age (weeks)				
Very preterm (22–27 weeks)	403 (0.5)	2247 (0.5)	591 (0.5)	2576 (0.4)
Moderately preterm (28–36 weeks)	4951 (6.2)	30,779 (6.6)	6481 (5.6)	31,701 (4.7)
Term (37–41 weeks)	66,354 (82.8)	367,439 (78.3)	98,033 (84.8)	552,939 (82.8)
Post term (≥ 42 weeks)	6454 (8.1)	49,734 (10.6)	6969 (6.0)	52,688 (7.9)
Missing	1957 (2.4)	18,784 (4.0)	3532 (3.1)	27,750 (4.2)

^a^ Information was drawn from the first child in multiple births; ^b^ Includes unmarried, single, divorced, separated, widowed, registered partner and other; ^c^ Quartiles drawn from each year; ^d^ Non-specific and gestational diabetes not included; ^e^ Maternal overweight and smoking include data from 2008 and 1999 onwards, respectively

The overall prevalence of stillbirth was slightly higher in migrant than in non-migrant women (migrants 0.56% vs non-migrants 0.49%, *p* < 0.001). However, the stillbirth prevalence was only higher in multiparous migrant women compared with the non-migrants (migrants 0.57% vs non-migrants 0.46%, p < 0.001), and not in the primiparous women (migrants 0.54% vs non-migrants 0.52%, *p* = 0.37). We found no difference between migrant and non-migrant women in whether the death of the infant had occurred before or after onset of labour. The time for the death of the infant for primiparous women was: migrants 89% before onset and 11% after onset vs non-migrants 87% before onset and 13% after onset (*p* = 0.26); and for multiparous women: migrants 87% before onset and 13% after onset vs non-migrants 90% before onset and 10% after onset (*p* = 0.11). However, information about the time of death was missing for 25% of all stillbirths (Additional file 1: Table S1).

**Fig. 2:**
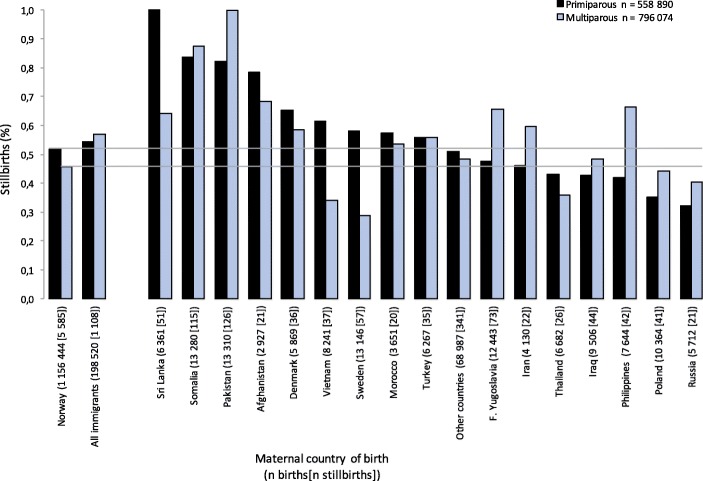
Prevalence of stillbirth in relation to maternal country of birth (N = 1,354,964) in Norway, 1990–2013. Maternal country of birth is presented with total number of births, and the number of stillbirths in brackets. The bars are sorted by the highest prevalence of stillbirths to multiparous women

**Fig. 3:**
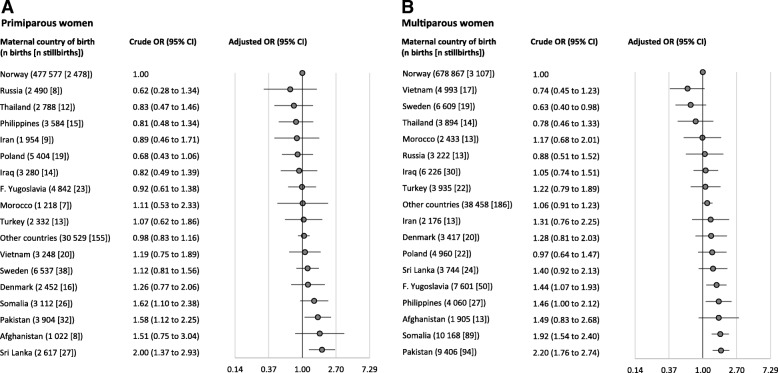
Associations between maternal country of birth and stillbirth in women giving birth in Norway, 1990–2013. Associations were estimated as odds ratios with 95% confidence intervals. The reference group was non-migrant women. All analyses were adjusted for year of birth, maternal age, marital status, consanguinity, level of education and income. Analyses of multiparous women were in addition adjusted for parity. Analyses for primiparous women in panel **a** and multiparous women in panel **b**. Abbreviations: OR, odds ratio; CI, confidence interval

The prevalence of stillbirth by maternal country of birth is shown in Fig. 2. In primiparous women the highest prevalence of stillbirth was found in women from Sri Lanka, Somalia and Pakistan, and in multiparous women in those from Pakistan, Somalia and Afghanistan. The lowest prevalence in primiparous women was found in women from Russia, Poland and the Philippines, and in multiparous women, in those from Sweden, Vietnam and Thailand. The crude and adjusted ORs for stillbirth in relation to maternal country of birth are shown in Fig. 3. In primiparous women (Fig. 3, panel A), there was an increased adjusted odds of stillbirth for women from Sri Lanka (aOR = 1.79; 95% CI 1.22–2.63) and Pakistan (aOR = 1.58; 95% CI 1.07–2.34), relative to non-migrant women. In multiparous women (Fig. 3, panel B), there was an increased adjusted odds of stillbirth for women from Pakistan (aOR = 1.71; 95% CI 1.34–2.18), Somalia (aOR = 1.67; 95% CI 1.30–2.16), the Philippines (aOR = 1.60; 95% CI 1.09–2.33), and Former Yugoslavia (aOR = 1.50; 95% CI 1.11–2.01), relative to non-migrant women.

The associations between stillbirth and other migration related factors, in terms of paternal origin, reason for immigration, length of residence in Norway and birthplace of firstborn child, are shown in Table 2. Primiparous migrant women whose babies were registered with a Norwegian-born father had a decreased adjusted odds of stillbirth (aOR = 0.73; 95% CI 0.58–0.93) compared to migrant women whose babies were registered with a foreign-born father. In contrast, and regardless of parity, migrant women with missing data on paternal origin had an increased adjusted odds of stillbirth (primiparous: aOR = 6.29; 95% CI 4.64–8.51; multiparous: aOR = 5.72; 95% CI 4.70–6.96).

**Table 2:** Migration related factors and their association with stillbirth in migrant women giving birth in Norway, 1990–2013

	Primiparous					Multiparous				
	n	Stillbirths	(%)	Crude OR (95% CI)	Adjusted^a^ OR (95% CI)	n	Stillbirths	(%)	Crude OR (95% CI)	Adjusted^b^ OR (95% CI)
Paternal origin										
Foreign-born (ref)	47,109	233	(0.5)	1.00	1.00	73,487	313	(0.4)	1.00	1.00
Norwegian-born	30,175	119	(0.4)	0.80 (0.63 to 1.00)	0.73 (0.58 to 0.93)	31,643	106	(0.3)	0.79 (0.63 to 0.98)	0.83 (0.65 to 1.05)
Missing ^c^	4029	90	(2.2)	4.60 (3.59 to 5.89)	6.29 (4.64 to 8.51)	12,077	247	(2.0)	4.88 (4.12 to 5.79)	5.72 (4.70 to 6.96)
Reason for immigration ^d^										
Nordic migrants (ref)		56	(0.6)	1.00	1.00	9145	37	(0.4)	1.00	1.00
Work/Education	13,410	46	(0.3)	0.54 (0.36 to 0.80)	0.58 (0.39 to 0.88)	9057	38	(0.4)	1.04 (0.66 to 1.64)	1.21 (0.76 to 1.92)
Family reunion or establishment	38,083	196	(0.5)	0.81 (0.60 to 1.09)	0.76 (0.55 to 1.06)	53,209	281	(0.5)	1.31 (0.92 to 1.86)	1.22 (0.85 to 1.74)
Refuge	9467	62	(0.7)	1.03 (0.71 to 1.48)	0.92 (0.62 to 1.37)	20,623	138	(0.7)	1.66 (1.14 to 2.41)	1.41 (0.95 to 2.11)
Length of residence										
< 2 years (ref)	30,801	148	(0.5)	1.00	1.00	18,029	99	(0.5)	1.00	1.00
2–5 years	28,095	158	(0.6)	1.17 (0.93 to 1.47)	1.20 (0.94 to 1.53)	42,549	221	(0.5)	0.95 (0.75 to 1.20)	0.97 (0.76 to 1.24)
6–9 years	10,299	64	(0.6)	1.30 (0.96 to 1.75)	1.28 (0.93 to 1.77)	27,294	160	(0.6)	1.07 (0.83 to 1.37)	1.04 (0.80 to 1.36)
≥ 10 years	12,118	72	(0.6)	1.24 (0.93 to 1.65)	1.27 (0.93 to 1.74)	29,335	186	(0.6)	1.16 (0.90 to 1.48)	1.06 (0.81 to 1.39)
Birthplace of firstborn child										
Norway (ref)						52,834	231	(0.4)	1.00	1.00
Other than Norway						40,483	267	(0.7)	1.51 (1.26 to 1.81)	1.28 (1.06 to 1.55)

^a^ Adjusted for year of birth, maternal age, marital status, consanguinity, level of education and income; ^b^ Adjusted for year of birth, parity, maternal age, marital status, consanguinity, level of education and income; ^c^ Missing data only refers to information on paternal country of birth, father can still be known by other variables in the data-set; ^d^ Missing data not included (primiparous: 1154 births, multiparous: 1283 births)

Primiparous women migrating for work or education had decreased odds of stillbirth compared to Nordic migrants (aOR = 0.58; CI 0.39–0.88), whereas multiparous refugees had a higher crude odds of stillbirth, relative to Nordic migrant women (Table 2). However, the finding in multiparous women did not reach statistical significance in adjusted regression analyses. Length of residence in Norway at the time of the index birth was not significantly associated with stillbirth in either crude or adjusted regression analysis.

Finally, multiparous migrant women who had given birth to their first child before arriving in Norway had higher odds of stillbirth in later births in Norway compared with multiparous migrant women who had their first child after arrival (aOR = 1.28; 95% CI 1.06–1.55).

## Discussion

### Main findings

This study has shown that the prevalence of stillbirth was slightly higher in migrant women compared to non-migrant women. Women from Pakistan, primiparous women from Sri-Lanka and multiparous women from Somalia, the Philippines and Former Yugoslavia were at highest risk of stillbirth. Babies with foreign-born fathers were associated with higher odds of stillbirth when compared to babies with Norwegian-born fathers, but only in births to primiparous women. Primiparous women migrating for work or education had decreased odds of stillbirth compared to Nordic migrants. Multiparous women who had given birth to their first child before immigration to Norway had an increased odds of stillbirth in later births, compared with multiparous migrant women who had their first child after immigrating. Stillbirth was not associated with length of residence.

### Strengths and limitations

This register study covers all births in Norway and the large sample size allowed for detailed analysis of women’s specific countries of birth and for separate analyses for primiparous and multiparous women. The inclusion of important migration related data, such as paternal origin, reason for immigration, length of residence and birthplace of firstborn child is unique and possible due to linkage between registers using personal identification numbers. Inclusion of these migration related factors led to a more complete analysis and added value to the interpretation of the data.

We did not adjust for maternal overweight or smoking as these variables were only available from 2008 and 1999, respectively. Overweight and smoking are well-documented risk factors for stillbirth, but are differently distributed in migrant women. Therefore, the observed differences in stillbirth between migrant and non-migrant women in our study might be stronger for women who represent countries with high rates of non-smokers, such as Afghanistan, Pakistan and Sri Lanka, and weaker for women from Poland and Former Yugoslavia with higher prevalence of smokers (data not shown).

Although we have adjusted for year of birth, this may not capture the full impact of changes in practice or background characteristics in sub-groups of both migrant and non-migrant women (i.e. increasing challenges with stillbirth risk factors such as overweight and diabetes [26]). The long time span of the study might therefore be seen as a limitation, also in relation to maternal country of birth as reasons for migration may have changed over the years, such as for migrant women from the Former Yugoslavia who fled the wars in the 1990s, while work or family reunion were more common reasons for immigration at other times [27].

### Interpretation

Our finding that migrant women overall had slightly higher odds of stillbirth compared with non-migrant women is consistent with findings from previous studies in Norway [28]. We also confirm the findings of others that migrant women constitute a heterogeneous group and stillbirth risk varied across maternal countries of birth [6, 7, 29]. Although migrant women from most countries had similar odds of stillbirth compared with non-migrant women, women from some countries did have an increased risk (Fig. 3).

Consistent with other studies [28–31], Pakistani women had the highest odds of stillbirth of all women. Consanguinity is a well-known risk factor for stillbirth and particularly high prevalence has been reported in Pakistani women [30, 31]. The increased odds remained statistically significant however, also after adjustment for consanguinity in the analysis. One possible explanation may be linked to repeated consanguinity in one or both parents’ families, which may increase the risk of perinatal loss [32]. Unfortunately, such information was lacking in the registers.

Women from Sri Lanka and Somalia also had higher risk of stillbirth in this as in other studies [6, 28, 29, 33–35]. The increased risk of stillbirth has previously been attributed to poorer health, malnutrition, consequences of flight from war and conflicts, lower attendance in antenatal care, communication difficulties, inequities in care provision [28] and for African migrant women, complications related to suboptimal care including delay in seeking health care and mothers refusing caesarean sections [35]. Somali women in particular tend to book late and make fewer visits for antenatal care [36]. We also showed that stillbirth risks were higher in multiparous women, but not in primiparous women from the Philippines and the Former Yugoslavia. This finding may be supported by previous literature. In particular, grand multipara Filipino women have previously been associated with an increased risk of type 2 diabetes [37], and type 2 diabetes is an important risk factor for stillbirth [38]. Future studies are warranted to confirm the robustness of these findings. These associations might have been present in other studies, but previous studies of stillbirth and maternal country of birth have not distinguished between primiparous and multiparous women [28, 29].

The higher odds of stillbirth when both parents were migrants compared with when fathers were Norwegian-born, is consistent with findings from one study from the US [39] and one from Canada [6]. In the Canadian study, especially foreign-born couples originating from a country with a high stillbirth rate, were at greater risk of stillbirth [6]. Couples in which both parents are migrants may have several disadvantages, particularly in terms of limited knowledge about the receiving country’s health care system, communication problems and access to equitable and individualised care [40]. However, the pathways between such disadvantages and stillbirth in migrant couples needs to be further investigated in order to improve maternity care for them.

Missing data on paternal origin was associated with increased odds of stillbirth. A Canadian study found that missing paternal information in general is a strong marker for increased risk of adverse birth outcomes [41]. One could speculate that missing information on paternal origin may be due to poor obstetric history taking from women, perhaps due to communication difficulties, or it may also offer important clues to caregivers related to the woman’s psychosocial environment. Additional studies are needed to elucidate the increased risk among migrant women with unknown information on fathers.

Multiparous migrant women who had given birth to their first child before arriving in Norway were at higher risk for stillbirth, compared with those who had their first child after arrival. According to the national guidelines, multiparous women with a previous normal pregnancy and birth were until 2005 regarded as low-risk in Norway and were recommended to have fewer antenatal care visits than primiparous women (seven vs eleven) [42]. The possible lack of important information about the first pregnancy in multiparous women with a first child born outside Norway, in combination with communication barriers and the practice of giving more limited attention to multiparous women, may possibly contribute to the increased risk of stillbirth in these women. For instance, preeclampsia, which is a leading cause of perinatal mortality worldwide, is associated with a 10-fold increased recurrent risk in a second pregnancy [43]. Our findings, therefore, suggest increased attention should be given to multiparous migrant women with a first child born outside Norway.

In a previous study from Sweden, the risk of stillbirth was higher in migrants who had been in Sweden for a short time period (< 5 years) compared with those who had been in Sweden for a longer period [44]. We found no such association in our study. In fact there was a tendency for an increased, although not statistically significant, risk of stillbirth with longer residence in the primiparous migrant group. Comparison of findings for migrants in general compared with host country-born women across studies may not be entirely appropriate however. Differences in maternal countries of origin among migrant groups in Sweden and Norway and therefore also in proportions of high risk groups may account for differences in findings. A better approach would be to compare study results by sub-groups of migrant women rather than the overall estimate for all migrant groups combined, as the association of length of residence with different health outcomes varies between sub-groups of migrants [8, 45]. Unfortunately, the numbers of stillbirths in our study were too few to perform such sub-group analyses.

We found decreased odds of stillbirth in primiparous women migrating for work or education compared to Nordic migrants. To our knowledge, this has not been reported before. One possible explanation could be the higher use of tobacco among Nordic women in our sample [46], an important risk factor for stillbirth [5, 47]. These findings need further investigation. Refugees, on the other hand, often constitute a particularly vulnerable socio-economic group post migration [48], and refugee background has been associated with a number of adverse pregnancy outcomes including stillbirth [7]. One review article describes similar diverging results between studies, which was interpreted as a matter of selection, as some refugees may be political refugees from more advantageous socioeconomic backgrounds and others are refugees fleeing from wars and conflicts [7]. Further, we did not include non-migrant women in our analysis on reason for immigration, and the diverging results may therefore be explained by a difference in the choice of reference group, as well as sample size.

## Conclusion

This study identifies sub-groups of migrant women who are at an increased risk of stillbirth, and highlights the need to improve care for them. Extra attention should be paid to women from certain countries, multiparous women who had their first baby before arrival and primiparous women whose babies have foreign-born fathers.

## Additional file

Additional file 1: **Table S1.** Time of stillbirth. (DOCX 17 kb)

## Abbreviations

aOR: Adjusted odds ratio
CI: Confidence interval
MBRN: The Medical Birth Registry of Norway
OR: Odds ratio
SSB: Statistics Norway
