# Supplementary material for: Stillbirth in relation to maternal country of birth and other migration related factors: a population-based study in Norway
Source: BMC Pregnancy Childbirth. 2019 Jan 5;19:5. doi: 10.1186/s12884-018-2140-3 (PMC6321699; doi:10.1186/s12884-018-2140-3)
Supplement: Supplementary file 1 — Table S1. Time of stillbirth. (DOCX 17 kb) [file 12884_2018_2140_MOESM1_ESM.docx]

| **Supplemental Table 1. Time of stillbirth.** | | | | | | | | | |
| --- | --- | --- | --- | --- | --- | --- | --- | --- | --- |
|  | **Primiparous women*** | | |  |  | **Multiparous women†** | | | |
|  | **Migrant** | | **Non-migrant** | |  | **Migrant** | | **Non-migrant** | |
|  | (n=442) |  | (n=2 478) |  |  | (n=666) |  | (n=3107) |  |
|  | *n (%)* | *(%)‡* | *n (%)* | *(%)‡* |  | *n (%)* | *(%)‡* | *n (%)* | *(%)‡* |
| Before onset | 299 (68) | (89) | 1 619 (65) | (87) |  | 451 (68) | (87) | 2 093 (67) | (90) |
| After onset | 37 (8) | (11) | 247 (10) | (13) |  | 66 (10) | (13) | 241 (8) | (10) |
| Missing | 106 (24) |  | 612 (25) |  |  | 149 (22) |  | 773 (25) |  |
| * p-value=0.26 | |  |  |  |  |  |  |  |  |
| † p-value=0.11 | |  |  |  |  |  |  |  |  |
| ‡ Percentage: non-missing data | | | |  |  |  |  |  |  |
